# Supplementary material for: Hypoxic conditioning in Parkinson’s disease: randomized controlled multiple N-of-1 trials
Source: Nat Commun. 2025 Sep 26;16:8469. doi: 10.1038/s41467-025-63324-2 (PMC12475152; doi:10.1038/s41467-025-63324-2)
Supplement: Supplementary file 1 — Supplementary Information [file 41467_2025_63324_MOESM1_ESM.pdf]

Supplementary materials

Supplementary Table 1: Inclusion and exclusion criteria

**Inclusion criteria**	**Exclusion criteria**
Clinical diagnosis of Parkinson's disease by a movement-disorder specialized neurologist	Individuals with diseases leading to restrictive and obstructive pulmonary diseases, pulmonary diffusion deficits, apnea and cardiac output deficits, such as pulmonary fibrosis, current smoking, current asthma, COPD, sleep apnea[1] or excessive alcoholic intake, arrythmia and congestive heart failure respectively
Hoehn and Yahr staging 1.5 to 3	Arterial blood gas abnormalities at screening day
Age > 18 years	Individuals with shortness of breath or other airway or breathing-related inconvenience related to lack of dopaminergic medication will be excluded
Participant can provide informed consent	Inability to comply to intervention in off-medication condition (for example due to extreme discomfort, distress or severe head tremor due to being OFF, i.e. without dopaminergic medication)
	Individuals with unstable dopaminergic medication dose (changes in the last month)
	Individuals likely to start dopaminergic treatment in the next month, also judged by their treating neurologist
	Individuals with active deep brain stimulation
	Individuals unable to provide informed consent

[1] *assessed by either confirmation through diagnosis by a somnologist or a self-reported positive history during the screening phase*

Primary outcomes

Supplementary Table 2: Lowest oxygen saturation (SpO_2) per participant. Green indicates greater or equal to lowest screening value, orange indicates lower SpO_2 during intervention than during screening.

**Participant**	**Lowest FiO_2 level**	**Lowest SpO2 during screening**	**Continuous** FiO_2 0.127/1.133	**Continuous** FiO_2 0.163	**Intermittent** FiO_2 0.127/0.133	**Intermittent** FiO_2 0.163
1	0.127	83%	82%	95%	85%	92%
2	0.127	79%	83%	93%	88%	94%
3	0.133	79%	85%	93%	88%	94%
4	0.127	83%	84%	94%	89%	94%
5	0.127	77%	86%	92%	86%	95%
6	0.127	80%	83%	92%	86%	93%
7	0.127	81%	84%	92%	85%	92%
8	0.127	87%	85%	94%	87%	94%
9	0.133	81%	82%	93%	89%	93%
10	0.133	85%	85%	94%	89%	94%
11	0.127	80%	84%	94%	92%	94%
12	0.133	81%	85%	93%	88%	93%
13	0.127	89%	83%	93%	90%	95%
14	0.127	82%	83%	93%	86%	93%
15	0.133	83%	85%	93%	90%	94%
16	0.127	86%	86%	94%	89%	95%
17	0.127	91%	87%	95%	90%	95%
18	0.133	86%	87%	93%	90%	93%
19	0.133	85%	85%	93%	89%	93%
20	0.133	88%	90%	95%	92%	97%

Supplementary Table 3: Stop criteria

Subjective parameters
- Dizziness, discomfort, and stress score higher than 7 on a 10-point Likert scale.

Vital parameters
- Systolic or diastolic blood pressure drop > 20 mmHg
- Heart rate > 140 bpm
- Breathing frequency > 25 per minute
- Oxygen saturation < 80%

Arterial blood gas (ABG) during screening
- pO2 < 40 mmHg (5,33 kPa)
- pCO2 < 25 mmHg (3,33 kPa)
- pH > 7,55

Other
- Occurrence of any other serious adverse event, or any other necessity to intervene for the participant's wellbeing as assessed by the supervising physician.

Supplementary Figure 1: Participant-reported dizziness, stress and discomfort during interventions on 10-point Likert scales. There were no between-intervention differences (P>0.05).

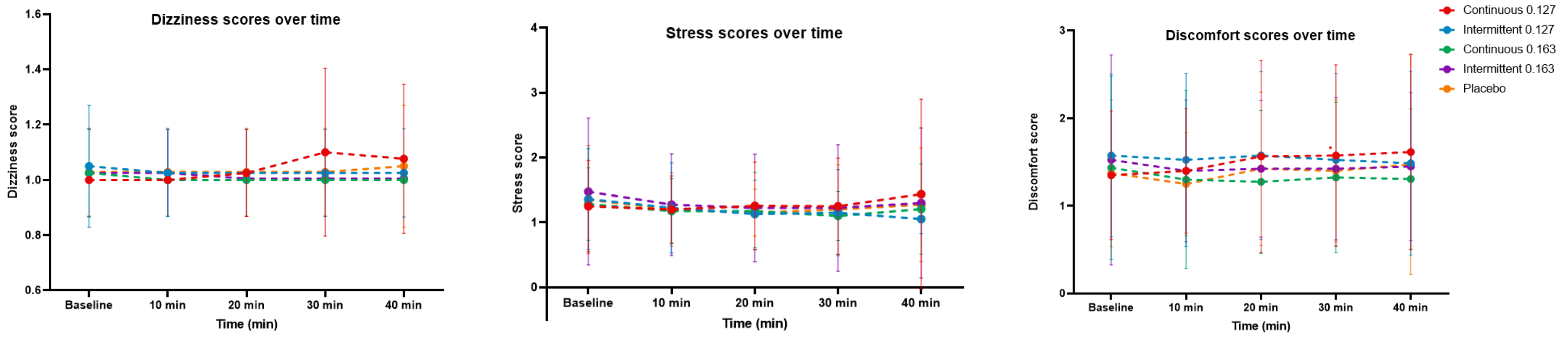

Supplementary Figure 2: Physiological parameters for the assessment of stop criteria during continuous hypoxic exposure: breathing frequency (A), heart rate (B), systolic blood pressure (C), and diastolic blood pressure (D). There were no significant between-intervention differences in physiological response (*P*>0.05).

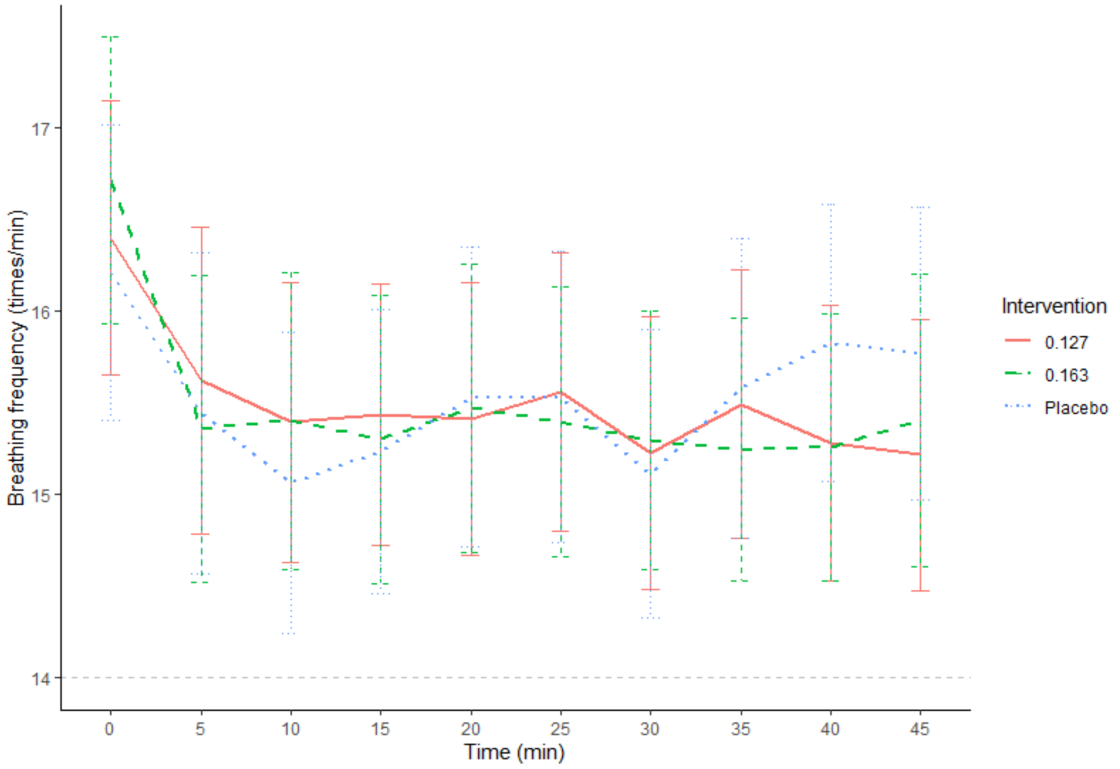

A

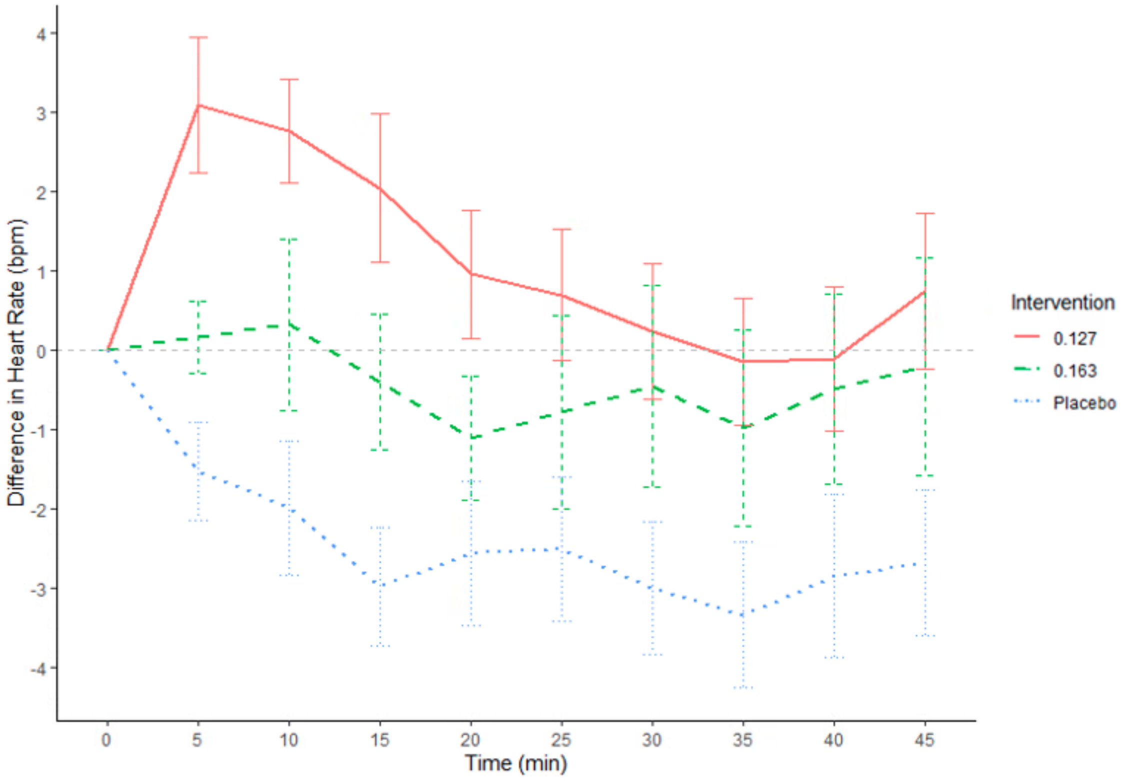

B

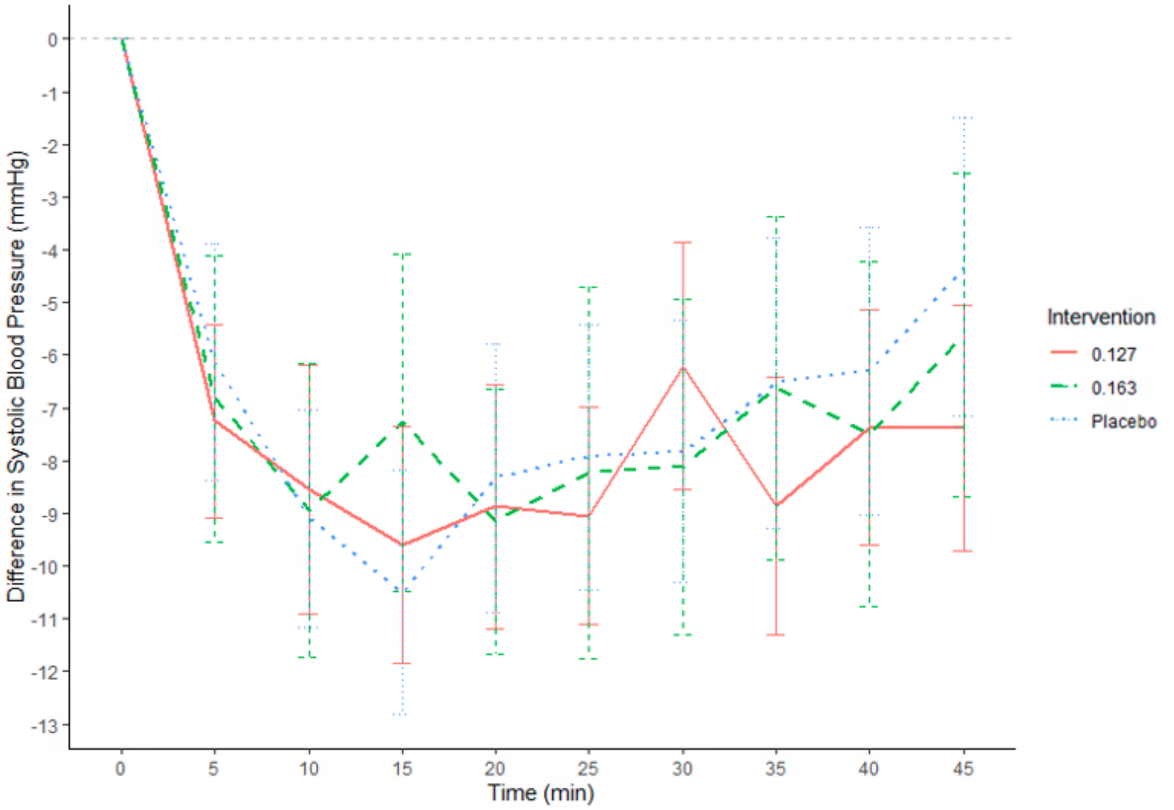

Difference in Systolic Blood Pressure (mmHg)
0
-1
-2
-3
-4
-5
-6
-7
-8
-9
-10
-11
-12
-13
0
5
10
15
20
25
30
35
40
45
Time (min)
Intervention
0.127
0.163
Placebo

C

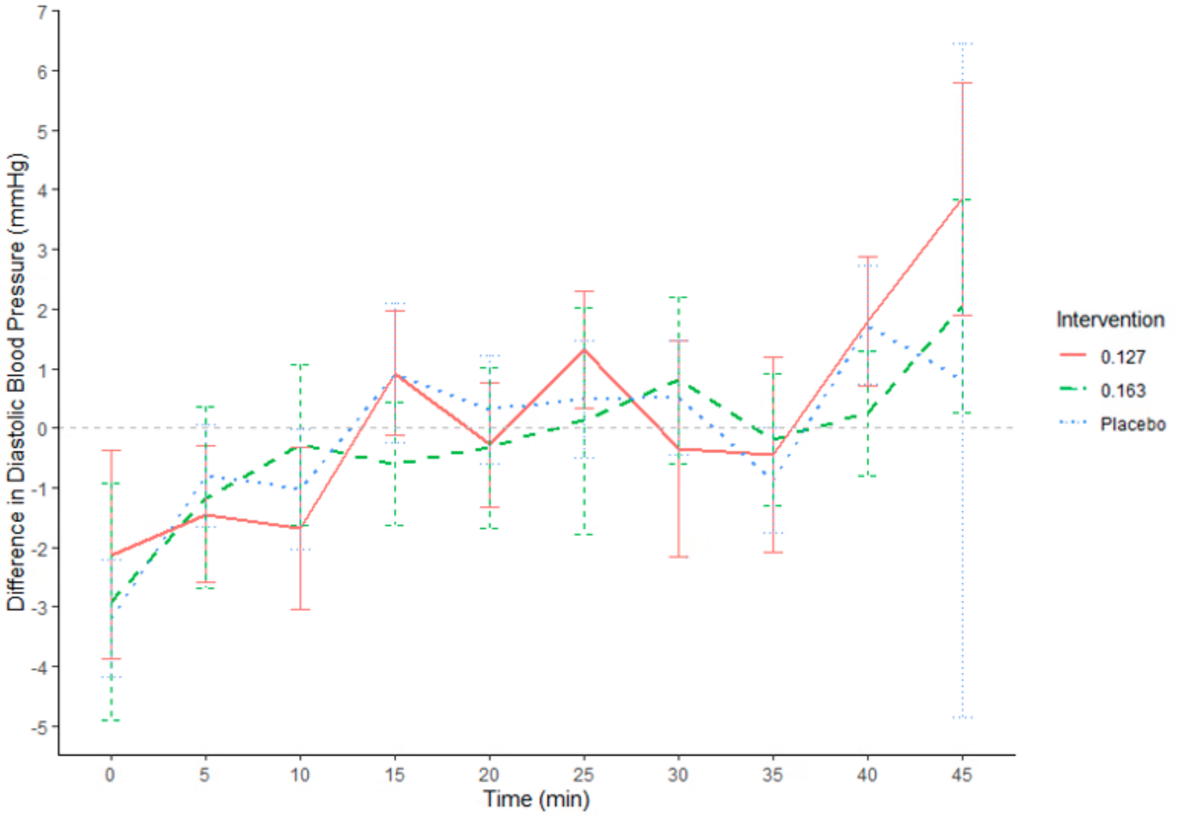

Difference in Diastolic Blood Pressure (mmHg)
7
6
5
4
3
2
1
0
-1
-2
-3
-4
-5
0
5
10
15
20
25
30
35
40
45
Time (min)
Intervention
0.127
0.163
Placebo

D

Secondary outcomes

Supplementary Figure 3A: linear mixed models for the three participant-rated symptoms**.**

Participant-selected symptom

Term	Estimate	Lower CI	Upper CI
(Intercept)	0.588	0.344	0.832
relevel(Treatment, ref = 5)1	0.246	-0.097	0.589
relevel(Treatment, ref = 5)2	0.253	-0.090	0.596
relevel(Treatment, ref = 5)3	0.574	0.229	0.919
relevel(Treatment, ref = 5)4	-0.114	-0.457	0.230
time	-0.095	-0.978	0.788
relevel(Treatment, ref = 5)1:time	0.055	-0.813	0.924
relevel(Treatment, ref = 5)2:time	0.170	-0.698	1.039
relevel(Treatment, ref = 5)3:time	-0.338	-1.212	0.536
relevel(Treatment, ref = 5)4:time	-0.177	-1.046	0.691

Urge to take dopaminergic medication

Term	Estimate	Lower CI	Upper CI
(Intercept)	0.297	0.032	0.561
relevel(Treatment, ref = 5)1	-0.167	-0.539	0.205
relevel(Treatment, ref = 5)2	0.053	-0.319	0.424
relevel(Treatment, ref = 5)3	0.483	0.109	0.856
relevel(Treatment, ref = 5)4	0.075	-0.296	0.447
time	-0.311	-1.320	0.699
relevel(Treatment, ref = 5)1:time	0.034	-0.907	0.974
relevel(Treatment, ref = 5)2:time	0.063	-0.878	1.004
relevel(Treatment, ref = 5)3:time	-0.213	-1.160	0.734
relevel(Treatment, ref = 5)4:time	-0.140	-1.080	0.801

Global symptom impression

Term	Estimate	Lower CI	Upper CI
(Intercept)	0.426	0.199	0.653
relevel(Treatment, ref = 5)1	-0.111	-0.430	0.209
relevel(Treatment, ref = 5)2	-0.119	-0.439	0.200
relevel(Treatment, ref = 5)3	0.253	-0.068	0.574
relevel(Treatment, ref = 5)4	-0.197	-0.517	0.123
time	-0.072	-0.796	0.651
relevel(Treatment, ref = 5)1:time	0.159	-0.650	0.968
relevel(Treatment, ref = 5)2:time	-0.017	-0.826	0.792
relevel(Treatment, ref = 5)3:time	-0.431	-1.245	0.383
relevel(Treatment, ref = 5)4:time	-0.431	-1.240	0.378

Legend:
1= continuous hypoxia F_IO_2 0.163
2= continuous hypoxia F_IO_2 0.127
3= intermittent hypoxia F_IO_2 0.163
4= intermittent hypoxia F_IO_2 0.127
5= placebo

Supplementary Figure 3A: linear mixed models for the three participant-rated symptoms with BDNF added as interaction factor**.**

Participant-selected symptom

Term	Estimate	Lower CI	Upper CI
(Intercept)	0.545	0.287	0.803
relevel(factor(Treatment), ref = 5)1	0.269	-0.092	0.629
relevel(factor(Treatment), ref = 5)2	0.241	-0.119	0.601
relevel(factor(Treatment), ref = 5)3	0.681	0.319	1.044
relevel(factor(Treatment), ref = 5)4	-0.226	-0.589	0.136
time	-0.167	-1.091	0.758
Delta BDNF concentration	-0.002	-0.017	0.013
relevel(factor(Treatment), ref = 5)1:time	0.106	-0.806	1.018
relevel(factor(Treatment), ref = 5)2:time	0.236	-0.677	1.149
relevel(factor(Treatment), ref = 5)3:time	-0.305	-1.223	0.614
relevel(factor(Treatment), ref = 5)4:time	-0.116	-1.035	0.802

Urge to take dopaminergic medication

Term	Estimate	Lower CI	Upper CI
(Intercept)	0.436	0.150	0.722
relevel(factor(Treatment), ref = 5)1	-0.297	-0.696	0.103
relevel(factor(Treatment), ref = 5)2	-0.066	-0.466	0.333
relevel(factor(Treatment), ref = 5)3	0.400	-0.002	0.802
relevel(factor(Treatment), ref = 5)4	-0.081	-0.484	0.321
time	-0.400	-1.462	0.663
Delta BDNF concentration	0.023	0.006	0.040
relevel(factor(Treatment), ref = 5)1:time	0.108	-0.904	1.120
relevel(factor(Treatment), ref = 5)2:time	0.137	-0.876	1.150
relevel(factor(Treatment), ref = 5)3:time	-0.140	-1.159	0.879
relevel(factor(Treatment), ref = 5)4:time	-0.077	-1.096	0.943

Global symptom impression

Term	Estimate	Lower CI	Upper CI
(Intercept)	0.492	0.245	0.739
relevel(factor(Treatment), ref = 5)1	-0.178	-0.523	0.167
relevel(factor(Treatment), ref = 5)2	-0.198	-0.543	0.147
relevel(factor(Treatment), ref = 5)3	0.212	-0.135	0.559
relevel(factor(Treatment), ref = 5)4	-0.306	-0.653	0.042
time	-0.157	-0.930	0.617
Delta BDNF concentration	-0.012	-0.026	0.003
relevel(factor(Treatment), ref = 5)1:time	0.243	-0.630	1.116
relevel(factor(Treatment), ref = 5)2:time	0.044	-0.830	0.918
relevel(factor(Treatment), ref = 5)3:time	-0.373	-1.252	0.505
relevel(factor(Treatment), ref = 5)4:time	-0.363	-1.243	0.516

Legend:

1= continuous hypoxia F_IO_2 0.163
2= continuous hypoxia F_IO_2 0.127
3= intermittent hypoxia F_IO_2 0.163
4= intermittent hypoxia F_IO_2 0.127
5= placebo

MDS-UPDRS

MDS-UPDRS is used to evaluate various aspects of Parkinson's disease including non-motor and motor experiences of daily living and motor complications. *Figure 3* shows the delta scores and is most pronounced for the intermittent hypoxia intervention at F_IO_2 0.163 (in purple). Despite not being a statistically significant improvement over placebo, the 3.5-point treatment effect of intermittent hypoxia 0.163 reaches the minimal clinically important difference (MCID) of 3.25 of the MDS-UPDRS part III score.

Supplementary Figure 4. Delta scores between baseline and post-intervention MDS-UPDRS part III. Lower score equals a better outcome.

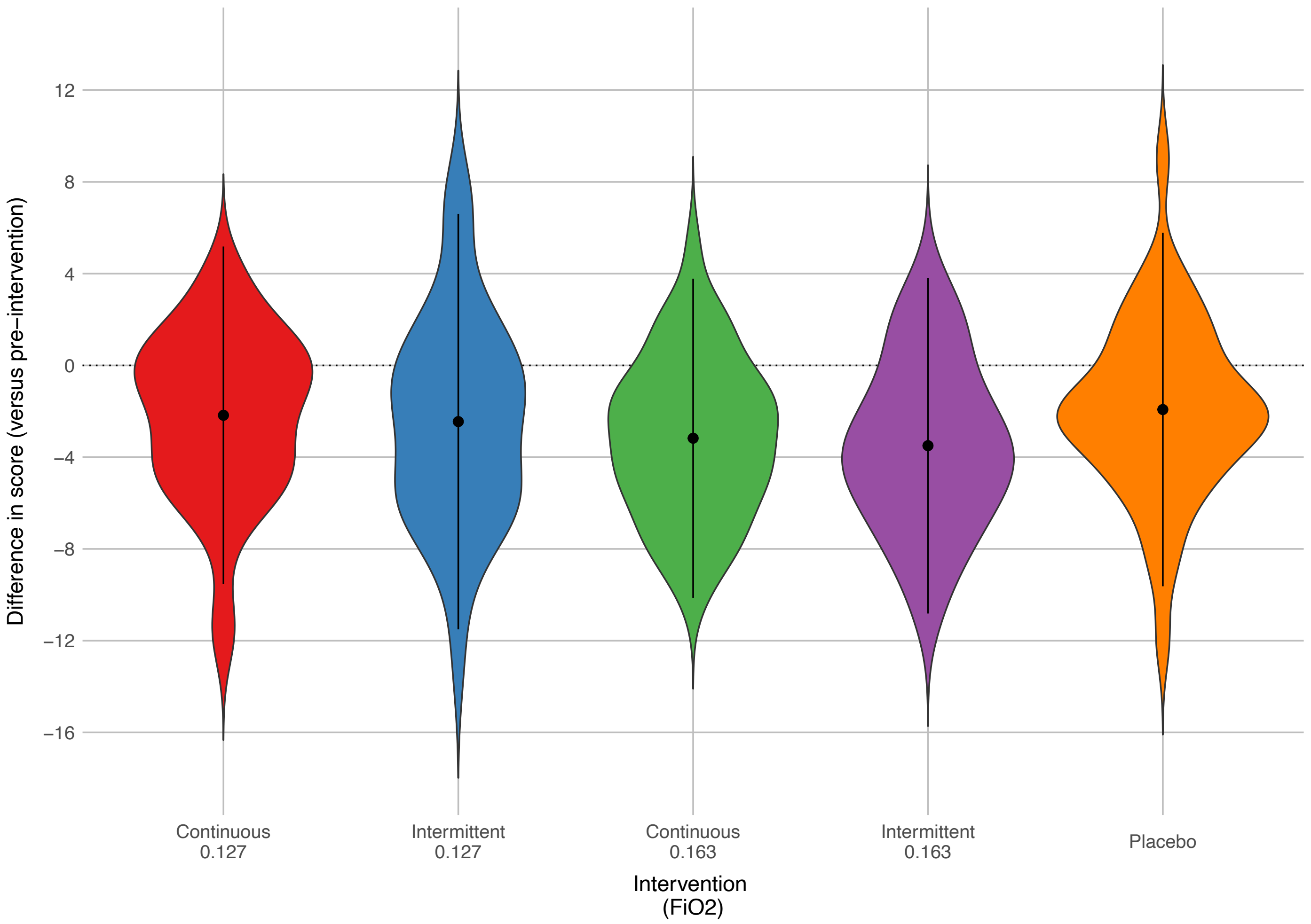

Mini Balance Evaluation Systems Test (MiniBEST)

MiniBEST tests balance, gait and mobility, including postural stability. There was no symptom worsening on the MiniBEST scores for any of the interventions. Although there was a trend towards a positive time effect across all interventions (*P*=0.059), there were no significant effect differences with placebo (*P*=0.98).

Supplementary Figure 5. Delta scores between baseline and post-intervention MiniBEST. Higher score equals a better outcome.

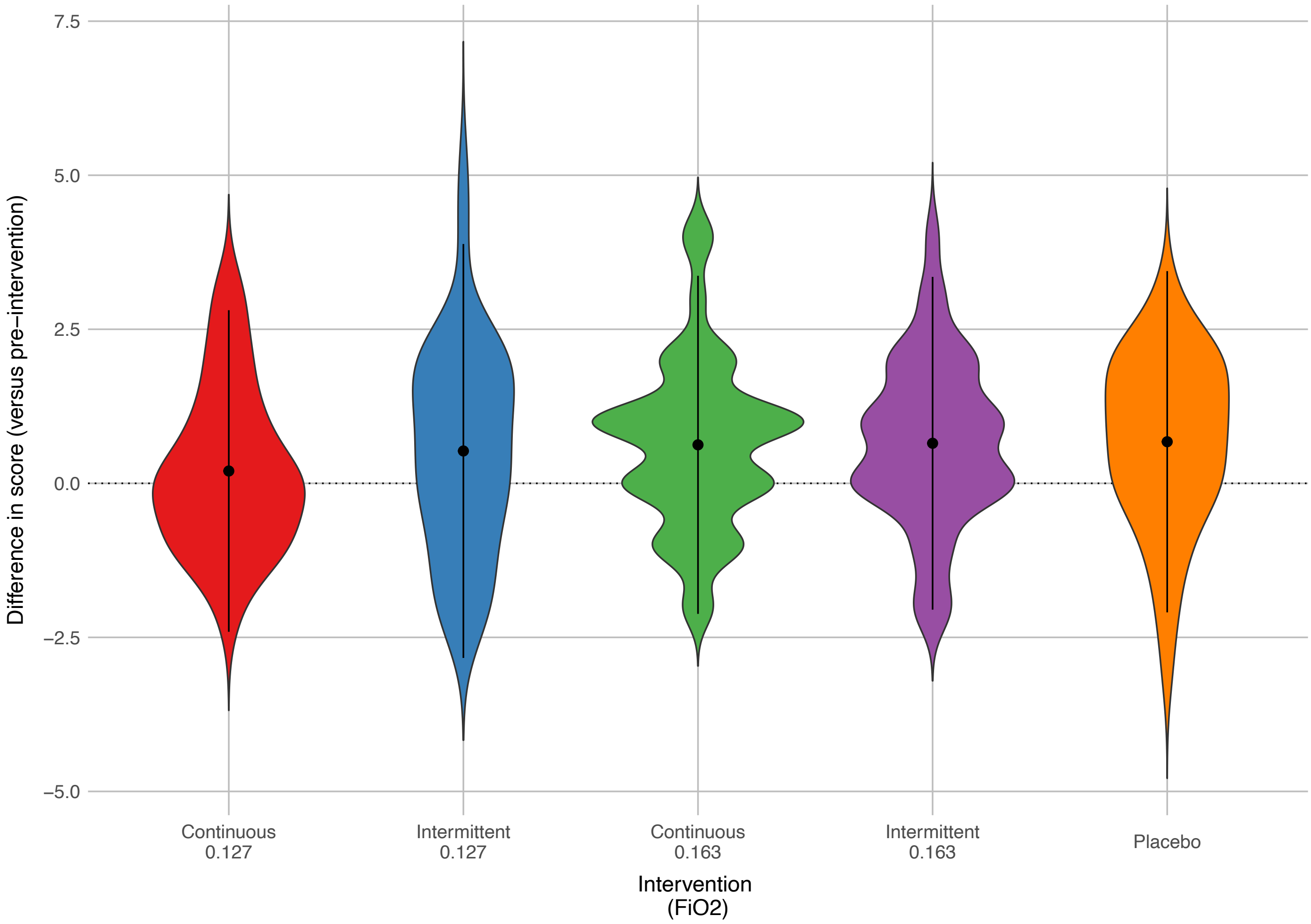

Purdue Pegboard Test (PPT)

PPT tests a broad range of abilities, including fine motor skills, hand-eye coordination and multitasking. It is used to objectify symptoms such as tremor and bradykinesia. There was no symptom worsening on either of these tests for any of the interventions. PPT did not show significant between-intervention differences (*P*=0.99).

Supplementary Figure 6. Delta scores between baseline and post-intervention PPT. Higher score equals a better outcome.

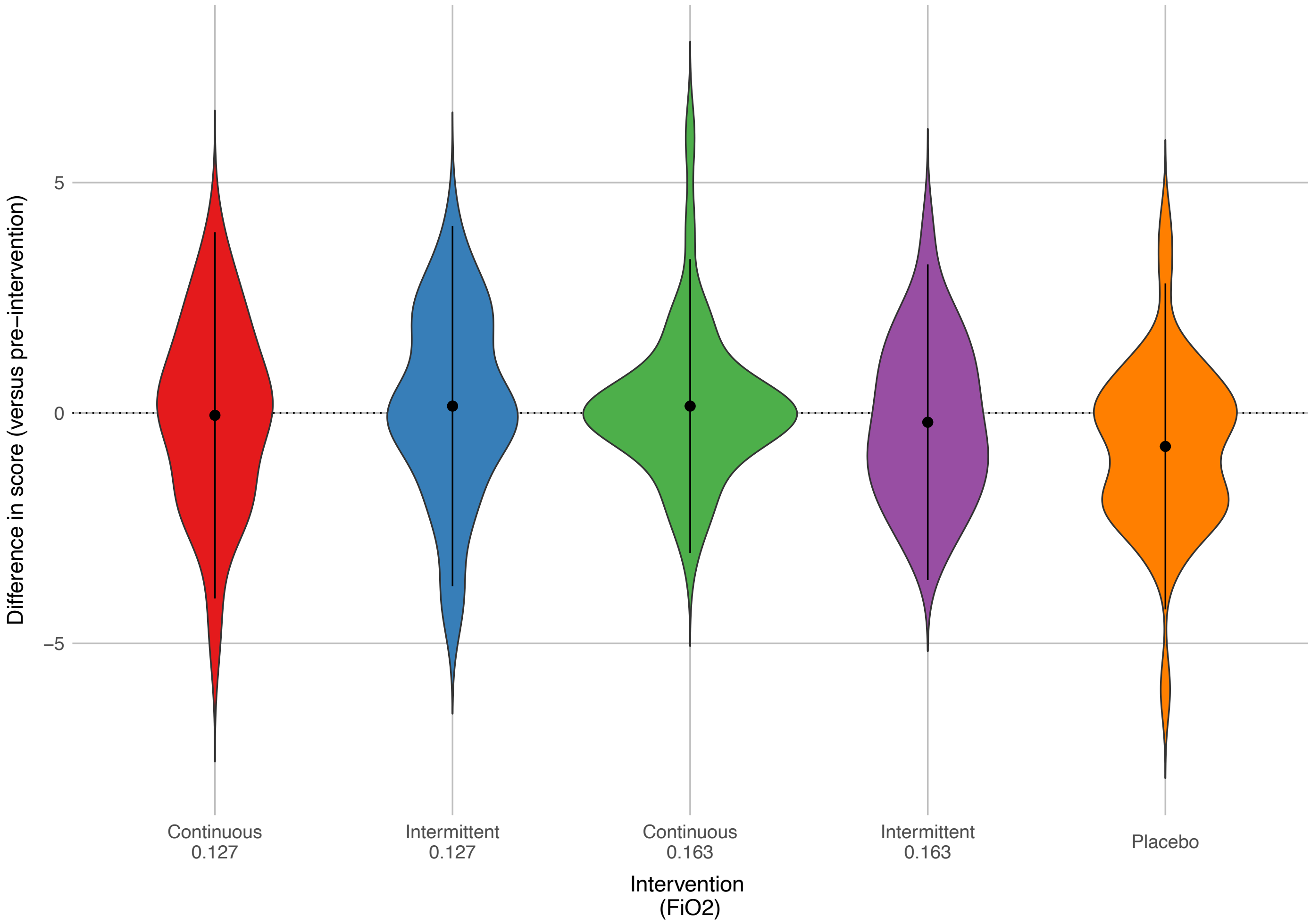

Timed Up & Go Test (TUGT)

TUGT tests walking speed, agility, balance and bradykinesia. There was no symptom worsening for any intervention. There were no significant between-intervention differences (*P*=0.32).

Supplementary Figure 7. Delta scores between baseline and post-intervention Timed Up & Go Test. A shorter time (lower score) indicates better mobility.

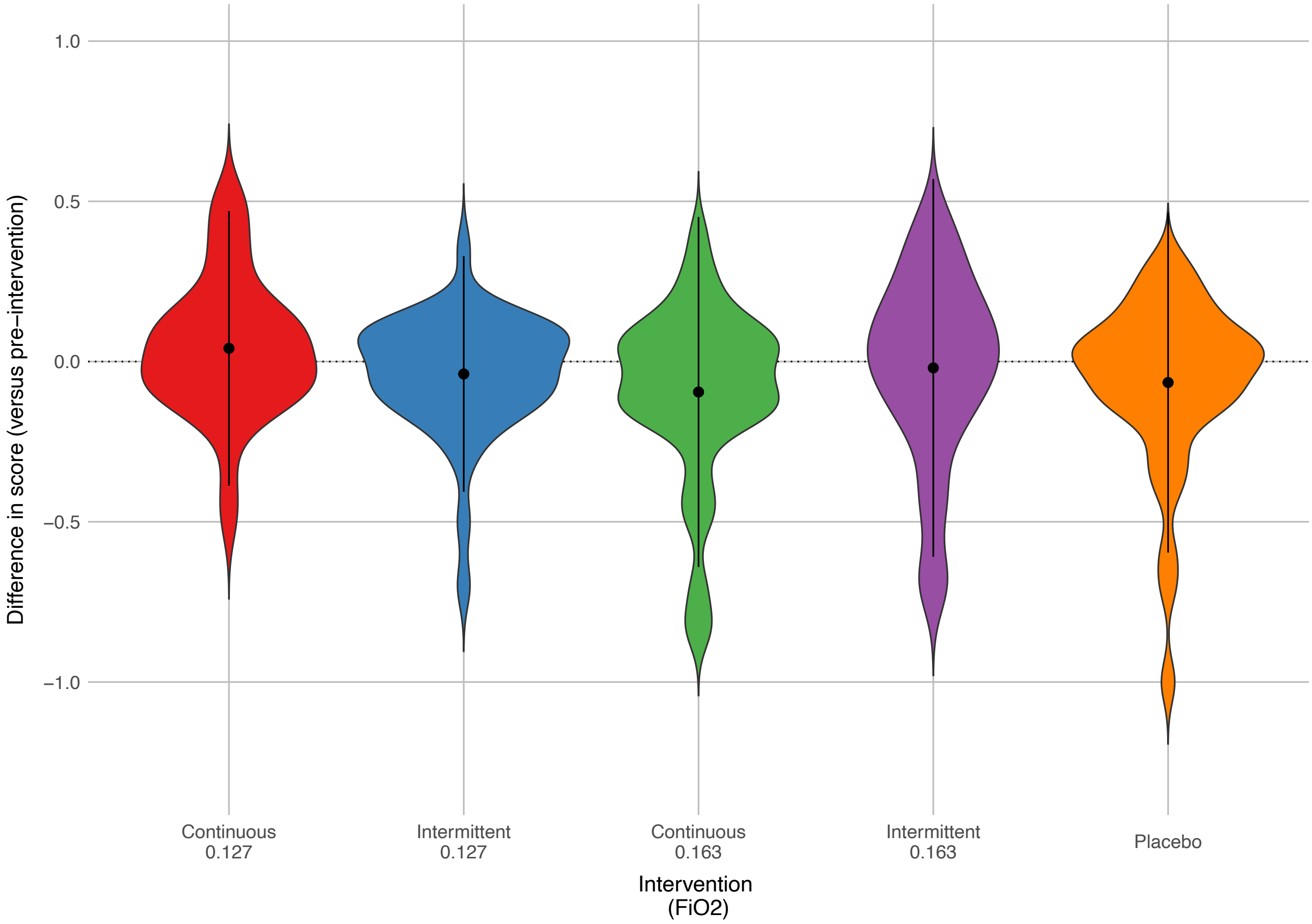

Exploratory outcomes

Supplementary Figure 8: Linear mixed model of PDGFrβ (A), EPO (B), cortisol (C), GFAP (D), NfL (E) and BDNF (F). For all markers, blood was drawn 30 minutes pre-intervention and 60 minutes post-intervention. An exception is cortisol, for which extra in-between measures were taken directly pre-intervention, directly after intervention and 30 minutes after the intervention to account for circadian rhythm effects.

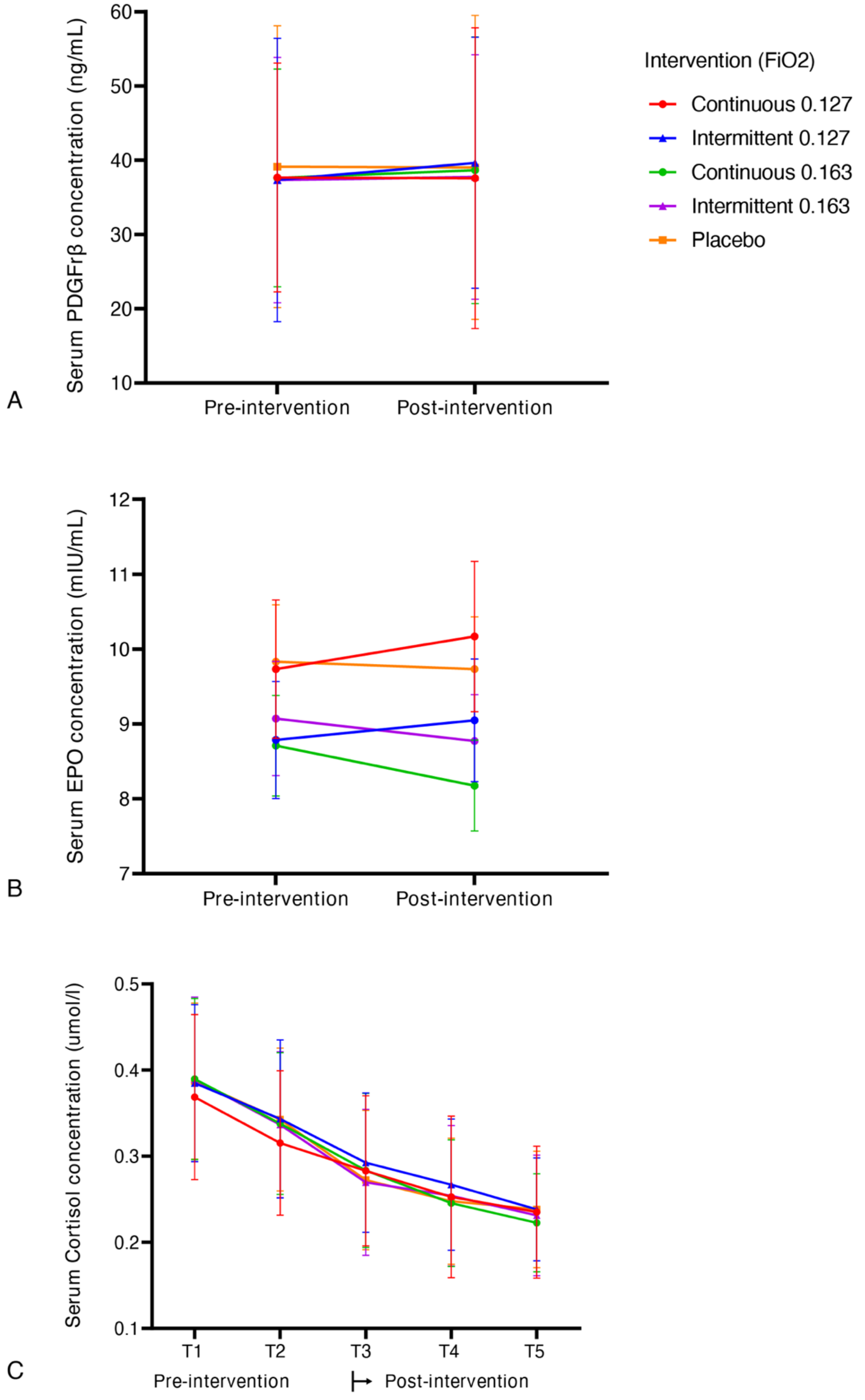

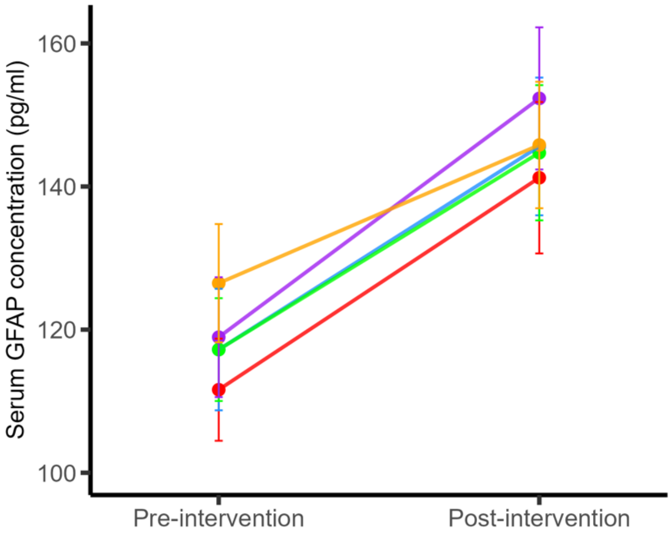
Serum GFAP concentration (pg/ml)
160
140
120
100
Pre-intervention
Post-intervention

D

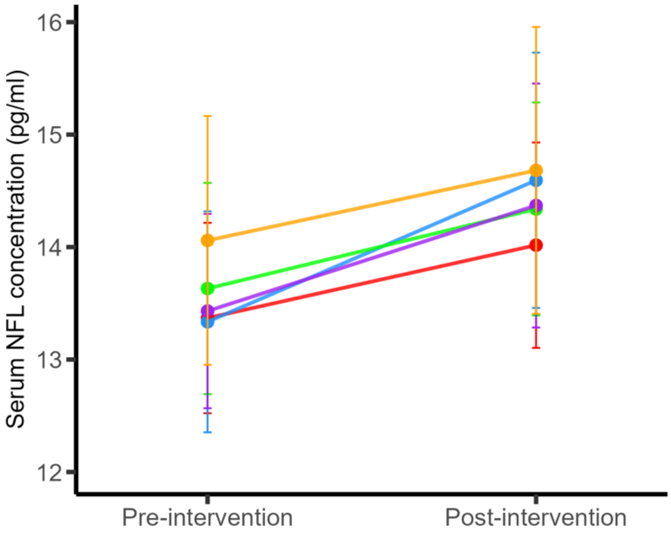
Serum NFL concentration (pg/ml)
16
15
14
13
12
Pre-intervention
Post-intervention

E

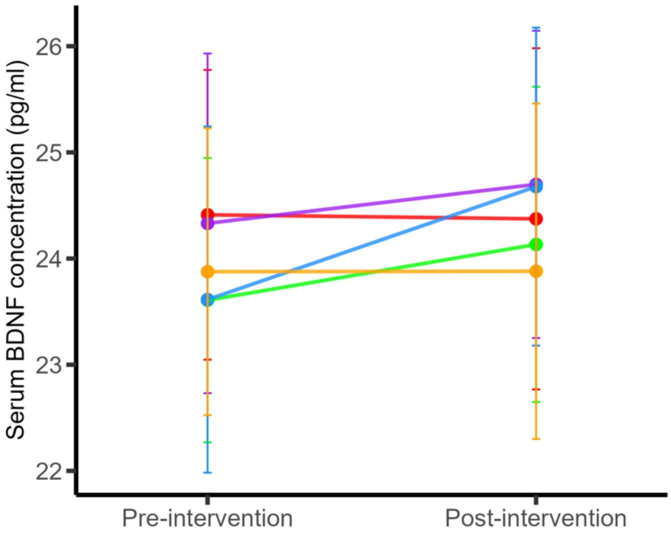
Serum BDNF concentration (pg/ml)
26
25
24
23
22
Pre-intervention
Post-intervention

F
