# Supplementary material for: Hypoxic conditioning in Parkinson’s disease: randomized controlled multiple N-of-1 trials
Source: Nat Commun. 2025 Sep 26;16:8469. doi: 10.1038/s41467-025-63324-2 (PMC12475152; doi:10.1038/s41467-025-63324-2)
Supplement: Supplementary file 2 — Reporting Summary [file 41467_2025_63324_MOESM2_ESM.pdf]

nature portfolio

Corresponding author(s): *Double-anonymous peer review submissions: write DAPR and your manuscript number here instead of author names.*

Last updated by author(s): *YYYY-MM-DD*

Reporting Summary

Nature Portfolio wishes to improve the reproducibility of the work that we publish. This form provides structure for consistency and transparency in reporting. For further information on Nature Portfolio policies, see our Editorial Policies and the Editorial Policy Checklist.

Statistics

For all statistical analyses, confirm that the following items are present in the figure legend, table legend, main text, or Methods section.

n/a	Confirmed	
☐	☒	The exact sample size (*n*) for each experimental group/condition, given as a discrete number and unit of measurement
☐	☒	A statement on whether measurements were taken from distinct samples or whether the same sample was measured repeatedly
☐	☒	The statistical test(s) used AND whether they are one- or two-sided<br>*Only common tests should be described solely by name; describe more complex techniques in the Methods section.*
☐	☒	A description of all covariates tested
☐	☒	A description of any assumptions or corrections, such as tests of normality and adjustment for multiple comparisons
☐	☒	A full description of the statistical parameters including central tendency (e.g. means) or other basic estimates (e.g. regression coefficient) AND variation (e.g. standard deviation) or associated estimates of uncertainty (e.g. confidence intervals)
☐	☒	For null hypothesis testing, the test statistic (e.g. *F*, *t*, *r*) with confidence intervals, effect sizes, degrees of freedom and *P* value noted<br>*Give P values as exact values whenever suitable.*
☐	☒	For Bayesian analysis, information on the choice of priors and Markov chain Monte Carlo settings
☐	☒	For hierarchical and complex designs, identification of the appropriate level for tests and full reporting of outcomes
☒	☐	Estimates of effect sizes (e.g. Cohen's *d*, Pearson's *r*), indicating how they were calculated

Our web collection on statistics for biologists contains articles on many of the points above.

Software and code

Policy information about availability of computer code

Data collection	There was no specific code used to obtain the data.
Data analysis	The complete code for both the hierarchical and Bayesian analysis of outcome measures is available through GitHub (https://github.com/Federica-Giardina/Talisman)

For manuscripts utilizing custom algorithms or software that are central to the research but not yet described in published literature, software must be made available to editors and reviewers. We strongly encourage code deposition in a community repository (e.g. GitHub). See the Nature Portfolio guidelines for submitting code & software for further information.

Data

Policy information about availability of data

All manuscripts must include a data availability statement. This statement should provide the following information, where applicable:

- Accession codes, unique identifiers, or web links for publicly available datasets
- A description of any restrictions on data availability
- For clinical datasets or third party data, please ensure that the statement adheres to our policy

Anonymized data is shared with The Michael J. Fox Foundation for Parkinson's Research (the study funder). This data may be kept for storage at a central repository either hosted by The Michael J. Fox Foundation, its collaborators, or consultants and will be kept indefinitely. Anonymized data will be made publicly available by the Foundation for the intended use of research in Parkinson's disease as well as other biomedical research studies that may not be related to Parkinson's disease.

Research involving human participants, their data, or biological material

Policy information about studies with human participants or human data. See also policy information about sex, gender (identity/presentation), and sexual orientation and race, ethnicity and racism.

Reporting on sex and gender	We consistently use the term 'sex' throughout the manuscript and report specific sex-based analyses.
Reporting on race, ethnicity, or other socially relevant groupings	We report the ethnicity of all particiipants and do not provide specific ethnicity-based analyses.
Population characteristics	We report in an extensive Table all demographic variables relevant to the study population and interpretation of the results.
Recruitment	Individuals with PD were recruited via the web-based ParkinsonNEXT recruitment website (www.parkinsonnext.nl). After a first telephone contact with the coordinating investigator, potential participants received an email with detailed information about the study, including an overview of necessary time investment and the risks of participation, as well as the informed consent form. Specifically, we inquired participants with a previous experience of positive effects of altitude about their symptoms.
Ethics oversight	Dutch medical-ethical committee for the eastern regions of The Netherlands (METC Oost-Nederland)

Note that full information on the approval of the study protocol must also be provided in the manuscript.

Field-specific reporting

Please select the one below that is the best fit for your research. If you are not sure, read the appropriate sections before making your selection.

☒ Life sciences ☐ Behavioural & social sciences ☐ Ecological, evolutionary & environmental sciences

For a reference copy of the document with all sections, see nature.com/documents/nr-reporting-summary-flat.pdf

Life sciences study design

All studies must disclose on these points even when the disclosure is negative.

Sample size	As this was the first-in-patient controlled trial into hypoxic conditioning in PD, the sample size was informed by a previous multiple N-of-1 trial experience from our center that also leveraged Bayesian analysis to analyze the effects of a symptomatic treatment (for a rare neuromuscular disease) and consensus for feasibility studies. We conducted a post-hoc sample size calculation and report this in the Discussion.
Data exclusions	No data were excluded.
Replication	All data and analytical methods are publicly available for replicability. Bayesian analysis leverages repeated measurements within any study participant to increase the likelihood that a measured effect is a real and reliable effect.
Randomization	Participants were equally divided in five groups with different interventions sequence according to a Latin square design with five 'periods' of interventions. These periods were grouped in two separate sets, each consisting of a randomized sequence with five periods (Figure 1). Randomization was conducted by a biostatistician (FG) using the R programming language.
Blinding	The lab technician was not blinded to the interventions for safety and monitoring purposes and oversaw randomization and ascertained intervention exposure. Participant and outcome assessor were blinded. Success of blinding was evaluated by asking participants to guess treatment assignment.

Reporting for specific materials, systems and methods

We require information from authors about some types of materials, experimental systems and methods used in many studies. Here, indicate whether each material, system or method listed is relevant to your study. If you are not sure if a list item applies to your research, read the appropriate section before selecting a response.

Materials & experimental systems

n/a	Involved in the study
☒	☐ Antibodies
☒	☐ Eukaryotic cell lines
☒	☐ Palaeontology and archaeology
☒	☐ Animals and other organisms
☐	☒ Clinical data
☒	☐ Dual use research of concern
☒	☐ Plants

Methods

n/a	Involved in the study
☒	☐ ChIP-seq
☒	☐ Flow cytometry
☒	☐ MRI-based neuroimaging

Clinical data

Policy information about clinical studies

All manuscripts should comply with the ICMJEguidelines for publication of clinical research and a completedCONSORT checklist must be included with all submissions.

Clinical trial registration	NCT05214287 (ClinicalTrials.gov)
Study protocol	The protocol is published through PMID: 35836147 (Janssen Daalen et al., 2022, BMC Neurology)
Data collection	Data were recorded at Radboud University Medical Center between February 2022 and May 2023. Interventions were administered in the hospital by an experienced lab technician, who also continuously monitors participants during and after the intervention. During the intervention, the inhaled FiO2 is measured continuously using the COSMED® metabolic system (Quark CPET metabolic cart for cardiopulmonary testing, COSMED Srl, The Metabolic Company, Italy), which also measures peripheral oxygen saturation, blood pressure, respiratory rate and tidal volume. CastorEDC, an electronic data capture system, was used to collect all data. In collaboration with our in-house Translational Metabolic Laboratory, serum analyses were performed.
Outcomes	We predefined all primary and secondary outcomes in our protocol which was also published (see before). Assessment of primary outcomes (safety and feasibility) was as follows:<br>Safety was assessed for three domains: 1) the number, nature and severity of adverse events, 2) participants meeting stop criteria during the intervention, and 3) participant-rated dizziness, discomfort and stress on a 10-point Likert scale (allowing half points) every 10 minutes during interventions.<br>The blinded assessor and main investigator rated AEs in terms of severity based on the Common Terminology Criteria for Adverse Events (CTCAE) version 5.083 and relatedness to study interventions or procedures. AEs were assigned to the last intervention before AE onset. In case of serious AEs, the treating physician (cardiologist or internist) of the participant was consulted for an independent assessment of relatedness to study interventions or procedures. During the intervention, participants were continuously monitored for meeting any stop criteria (Supplementary Materials).<br>Feasibility was assessed by a customized questionnaire after session one, five and ten (i.e., study completion). The questionnaire was informed by a widely used feasibility framework 84. Items in each category (e.g., acceptability, expectancy) were subsequently inspired by a variety of healthcare feasibility questionnaires.<br><br>Secondary outcomes were analyzed as follows:<br>Acute effects on PD symptoms were analyzed by participant-reported and assessor-rated standardized motor scales that were measured 30 minutes pre- and 30 minutes post-intervention, unless indicated otherwise. This means that before every intervention, a novel baseline measurement of secondary and explorative outcomes was conducted. The post-intervention window overlaps with the first therapeutic window of conditioning effects.<br>Participant-reported motor outcomes included 10-point Likert scales (allowing half points) for an important participant-selected symptom, general symptom impression and the urge to take dopaminergic medication. The participant-selected symptom category was based on goal attainment scaling principles – the symptom had to be prominent to the participant, fluctuate to some extent, and changes in severity had to be conveniently apparent to the participant. Participants scored these scales pre-intervention, and after 30 minutes, hourly in the five hours following intervention, and one, two and three days after the intervention in the morning, when the second window of hypoxic conditioning wanes.<br>Assessor-rated scales in the OFF-state consisted of MDS-UPDRS-III, Purdue pegboard test, Timed Up & Go Test and the Mini Balance Evaluation Systems Test (MiniBEST). Additionally, accelerometry-measured resting tremor (Move4, movisens GmbH, Germany) was measured pre-intervention, during intervention, directly after intervention and 30 minutes after intervention.

Plants

Seed stocks	N/A
Novel plant genotypes	N/A
Authentication	N/A
